# Modelling the economic impact of substandard uterotonics on postpartum haemorrhage in Nigeria: safeguarding medicine quality can reduce costs and contribute towards universal health coverage

**DOI:** 10.1136/bmjph-2023-000624

**Published:** 2025-04-05

**Authors:** Yi-Fang Ashley Lee, Colleen R Higgins, Petra Procter, Sara Rushwan, Chimezie Anyakora, Ahmet Metin Gülmezoglu, Lester Chinery, Sachiko Ozawa

**Affiliations:** 1 Division of Practice Advancement and Clinical Education, The University of North Carolina at Chapel Hill Eshelman School of Pharmacy, Chapel Hill, North Carolina, USA; 2 Concept Foundation, Geneva, Switzerland; 3 School of Science and Technology, Pan-Atlantic University, Lagos, Nigeria; 4 Bloom Public Health, Abuja, Nigeria; 5 Department of Maternal Child Health, The University of North Carolina at Chapel Hill Gillings School of Global Public Health, Chapel Hill, North Carolina, USA

**Keywords:** Public Health, Public Health Practice, economics, Female

## Abstract

**Introduction:**

Little evidence exists on the economic threat that substandard uterotonics pose to postpartum haemorrhage (PPH), maternal mortality, and national health systems. For clinical emergencies such as PPH, the quality of the uterotonic drugs required for prevention and treatment plays a central role in whether a severe outcome or fatality occurs and has a direct knock-on effect on the cost of further treatment and care. We modelled the health and economic burden of substandard uterotonics on PPH in Nigeria.

**Methods:**

A decision-tree model was built to simulate women giving birth in various healthcare settings, using uterotonics of varying quality, and facing PPH risks. We used the Demographic and Health Survey for care-seeking data and the Cochrane review for uterotonic effectiveness. Trial data from the Early detection of postpartum haemorrhage and treatment using the WHO MOTIVE bundle (E-MOTIVE) was applied for health outcomes by oxytocin quality. Scenarios were compared with and without substandard uterotonics.

**Results:**

We estimated that using substandard uterotonics led to avertable out-of-pocket and productivity losses totaling US$89 million (~₦ 68.4 billion) annually in Nigeria. These avertable losses were the result of using substandard uterotonics in 1.6 million mothers. Without substandard uterotonics, healthcare providers can avert nearly 75 000 preventable PPH cases, reduce uterotonic use, save blood transfusions and avert around 1500 maternal deaths due to PPH annually in Nigeria.

**Conclusion:**

This study demonstrates that use of quality-assured uterotonics would result in substantial reductions in the economic and health burden of PPH and contribute to decreasing maternal mortality and morbidity. Use of substandard uterotonics leads to increased out-of-pocket expenses and costs to health systems, which should be prevented to promote universal health coverage (UHC). Medicines quality assurance improves health outcomes and results in cost savings for governments to scale their implementation of UHC.

**WHAT IS ALREADY KNOWN ON THIS TOPIC**

- Maternal mortality and morbidity in low- and middle-income countries (LMICs) resulting from postpartum haemorrhage (PPH) could be significantly reduced through the effective use of quality-assured preventative uterotonics during the third stage of labour.
- Studies have shown the presence of substandard quality maternal health medicines in LMIC markets, including in Nigeria.
- Each instance of medicine failure or adverse reaction incurs costs to the patient, the healthcare system and payors responsible for financing healthcare.
- Studies have linked a lack of progress towards meeting universal health coverage (UHC) goals with prevalence of substandard medicines in LMICs.

**WHAT THIS STUDY ADDS**

- We quantify the clinical outcomes and costs of PPH in the context of universal health coverage (UHC) in Nigeria and estimate the costs and outcomes attributable to substandard uterotonics.
- Substandard uterotonics not only adversely affect the health of mothers and their families, but they also impose avoidable costs on the health system, which could otherwise be redirected to more productive investments.
- Our findings emphasise the significance of providing mothers with access to a range of quality-assured uterotonics for preventing PPH.

**HOW THIS STUDY MIGHT AFFECT RESEARCH, PRACTICE OR POLICY**

- An investment in quality-assured uterotonics should be considered an investment in UHC by reducing unnecessary out-of-pocket expenses and costs to health systems.
- The wide availability of substandard medicines is impeding LMIC countries’ progress towards sustainable development goals and UHC.
- Health ministries need to strengthen governance of medicines procurement systems and invest in cold chain infrastructure, as well as ensure that procurers are financially able and empowered to purchase quality-assured medicines as part of standard treatment guidelines that have been implemented at all levels of the health system.

## Introduction

 Postpartum haemorrhage (PPH) is the leading cause of maternal deaths globally, affecting an estimated 14 million women who give birth each year and accounting for more than 70 000 maternal deaths annually.^1 2^ The majority of maternal deaths from PPH occur in low- and middle-income countries (LMICs), and two-thirds of maternal deaths take place in sub-Saharan Africa.^1^ Specifically in Nigeria, PPH represents up to 44% of maternal mortality.^3^ Nigeria has one of the highest maternal mortality ratios (1047 maternal deaths per 100 000 live births), accounting for 81 747 maternal deaths in 2020, most of which could have been prevented. Prophylactic uterotonic medications such as oxytocin and misoprostol are recommended by the WHO and professional bodies globally to prevent PPH during the third stage of labour.^1 4^ Oxytocin and misoprostol are widely available in developing countries. However, previous studies have documented that nearly half of the uterotonic samples tested in LMICs failed quality tests.^5 6^ A 2018 study found a failure rate in Nigeria of 74% and 33% of oxytocin and misoprostol samples, respectively.^7^ Moreover, healthcare is often paid out-of-pocket (OOP) in Nigeria, putting mothers and their households under financial burden^8^ in an already resource-constrained environment. Continued lack of access to quality-assured uterotonics threatens Nigeria’s progress towards universal health coverage (UHC) goals.

For clinical emergencies such as PPH, the quality of the uterotonic drugs required for prevention and treatment of PPH plays a central role in whether a severe outcome or fatality occurs and has a direct knock-on effect on the cost of further treatment and care.^9^ Substandard medicines are ‘authorised medical products that fail to meet either their quality standards or specifications, or both.’^10^ Studies have been undertaken on the quality of maternal health medicines to assess the prevalence and severity of substandard medicines in LMIC markets.^11^,^17^ However, this evidence has led to little change in health policy at the national level in LMICs.^11^ Quantitative estimates of the impact of substandard medicines have been limited to date, but are urgently needed to make a stronger economic argument for safeguarding medicine quality throughout health systems in LMICs.

When a medicine’s quality is not safeguarded, the impact is both harmful and costly. Every medicine failure or adverse reaction bears a cost to the patient, the health system and the payors financing healthcare.^18^ In many LMICs, the use of substandard medicines is widespread and adds a significant burden to health systems that are already under strain from a high burden of disease, lack of financing, and weak regulatory systems.^19^ A 2019 study was the first to present a framework making a link between medicines quality and UHC, a global target for all individuals and communities to receive health services they need without suffering financial hardship.^20^ Taking a systems mapping approach, the framework highlights the numerous outcomes that can be associated with use of substandard medicines and how each relates to economic loss or waste that prevents countries from reaching UHC within a health system.^18 19^ This study builds on this framework in an effort to quantify the linkage between uterotonic quality and UHC.

The objective of this study is to highlight the importance of safeguarding uterotonic quality in LMICs using Nigeria as a use case, in the context of UHC and the United Nations’ (UN) Sustainable Development Goals (SDGs). This work focuses on SDG 3.1, to reduce the global maternal mortality ratio to less than 70 maternal deaths per 100 000 live births by 2030, and SDG 3.8, to provide access to quality essential healthcare services without financial risk, as well as ensure access to safe, effective, quality and affordable essential medicines and vaccines for all.^21^ This study demonstrates the health and economic impact of substandard uterotonics and their burden on governments, healthcare providers, and families.

## Methods

We adopted a decision tree model to assess the health and economic impact of substandard uterotonics in Nigeria.^22^ The model was developed using Python (V.3.8). The model simulates Nigerian pregnant women giving birth in various settings, the use of uterotonics, clinical outcomes, and associated costs. Model inputs were extracted from available literature and key informant insights. The primary outputs for the model are estimates of direct costs and productivity losses based on health outcomes.

### Model inputs

A targeted literature review was performed to inform our model inputs. Searches were conducted primarily through PubMed and supplemented by Google and ISI Web of Knowledge. We used iterations of search terms such as “uterotonics,” “uterotonics quality,” “maternal mortality,” “postpartum hemorrhage,” “utilization,” “costs,” and “Nigeria.” We also assessed institutional websites of the WHO, the World Bank and UN, and analysed Nigeria’s latest Demographic and Health Survey (DHS) data.^23^

Table 1 and online supplemental table S1 and S2 present key data inputs for our model. We used population characteristics from DHS, including age, rurality, region, wealth quintile, and mothers’ education.^23^ The proportions of care-seeking locations and the delivery methods were extracted from DHS.^23^ We modelled women giving birth through vaginal or caesarean section (c-section) delivery at four types of locations: (1) public hospitals (vaginal and c-section), (2) public primary health centres (PHCs) (vaginal), (3) private hospitals (vaginal and c-section) and (4) homes (vaginal). The likelihood of receiving substandard uterotonics was estimated using the prevalence of failed uterotonics found through quality testing, which was different for public and private sectors.^7^

**Table 1:** Key model inputs and sources

Parameter variable (unit)	Value (uncertainty range)		Source
Demographics			
Total population	213 401 323		
Birth rate (per 1000 people)	37		United Nations^4^
Maternal mortality rate (per 100 000 live births)	1047		WHO^1^
Life expectancy at birth, female (years)	53		United Nations
Mean age at maternal death (years)	30.8		Olamijulo *et al*^36^
GDP per capita (US$)	2066		World Bank^5^
Mothers' location			
Region	North	South	
Urban (%)	27	64	Demographic and Health Survey^23^
Rural (%)	73	36	
Care-seeking locations and birth methods by mother’s characteristics (%)			
Region	North	South	Demographic and Health Survey^23^
Public hospital and vaginal birth	19	37	
Public hospital and c-section	1	2	
Public PHC and vaginal birth	1	1	
Private hospital and vaginal birth	4	27	
Private hospital and c-section	0	4	
Home	75	30	
Proportion diagnosed with PPH (%)	30		Wakili *et al*^24^ and Sotunsa *et al*^34^
Proportion referred among severe PPH cases from home and PHC (%)	24		Banke-Thomas *et al*^6^
Utilisation of uterotonics (%)	Facility	Home	
Oxytocin	26	0	Ejekam *et al*^31^ and Gallos *et al*^9^
Oxytocin+misoprostol	52	0	
No uterotonics given	22	100	
Proportion of substandard uterotonics (%)	Public sectors	Private sectors	
Oxytocin	80	72	Anyakora *et al*^7^
Misoprostol	38	32	
Oxytocin dose used by medicine quality	Quality oxytocin	Substandard oxytocin	
10 IU	99.6%	69.2%	Gallos *et al*^9^
20 IU	0.0%	29.8%	
30 IU	0.4%	1.0%	
Risk of health outcomes			
Oxytocin	Vaginal birth	C-section	
PPH≥500 mL	0.12 (0.10–0.15)	0.6 (0.57–0.63)	Gallos *et al*^9^
PPH≥1000 mL	0.03 (0.02–0.04)	0.13 (0.13–0.14)	
Oxytocin+misoprostol	Vaginal birth	C-section	
PPH≥500 mL	0.09 (0.07–0.11)	0.42 (0.35–0.52)	
PPH≥1000 mL	0.03 (0.02–0.03)	0.12 (0.09–0.13)	
Misoprostol	Vaginal birth		
PPH≥500 mL	0.13 (0.12–0.15)		
PPH≥1000 mL	0.04 (0.03–0.04)		
Heat-stable Carbetocin	Vaginal birth	C-section	
PPH≥500 mL	0.09 (0.08–0.10)	0.44 (0.39–0.48)	
PPH≥1000 mL	0.03 (0.02–0.03)	0.12 (0.10–0.13)	
No prophylactic uterotonics	Vaginal birth		
PPH≥500 mL	0.24 (0.19–0.29)		
PPH≥1000 mL	0.05 (0.04–0.06)		
Risk ratio of PPH for substandard uterotonics			
PPH≥500 mL	1.29		Gallos *et al*^9^
PPH≥1000 mL	1.26		
Proportion of women receiving additional uterotonic treatments	Quality uterotonics	Substandard uterotonics	
Among diagnosed PPH cases (%)	70	89	Gallos *et al*^9^*
Among undiagnosed PPH cases (%)	35	44	Assumption†
Proportion of blood transfusions			
Among diagnosed PPH cases (%)	15	47	Gallos *et al*^9^
Among undiagnosed PPH cases (%)	8	23	Assumption†
Proportion of postpartum surgeries			
Among vaginal births with severe PPH	20		KOL Assumption
Out-of-pocket costs (USD)			
Public hospital	North	South	
Vaginal birth			Cost data were collected from 32 facilities by key informants.
No PPH	39.3 (0–78.6)	176.85 (9.91–314.4)	
Mild PPH	31.44 (15.72–188)	275.1 (17.72–314.4)	
Severe PPH without surgery	124.45 (52.4–196.5)	340.6 (157.2–524)	
Severe PPH with surgery	255.45 (117.9–393)	484.7 (314.4–655)	
C-section			
No PPH	176.85 (39.3–314.4)	347.15 (39.3–655)	
Mild PPH	275.1 (78.6–471.6)	432.3 (78.6–786)	
Severe PPH with surgery	350.43 (91.7–655)	481.43 (91.7–917)	
Primary health centre			
Vaginal birth			
No PPH	13.1 (0–26.2)	176.85 (91.7–262)	
Mild PPH	26.06 (4.95–47.16)	216.15 (117.9–314.4)	
Severe PPH without surgery	78.6 (39.3–117.9)	275.1 (157.2–393)	
Private hospital			
Vaginal birth			
No PPH	362.29 (69.58–655)	432.3 (3.3–655)	
Mild PPH	545.63 (43.27–1048)	681.2 (277.96–1048)	
Severe PPH without surgery	702.67 (95.33–1310)	851.5 (393–1310)	
Severe PPH with surgery	1109 (253–1965)	1179 (393–1965)	
C-section			
No PPH	1205 (314.4–2096)	1205 (6.59–2096)	
Mild PPH	1216 (73.33–2358)	1441 (395.86–2358)	
Severe PPH with surgery	2544 (128.33–6550)	2849 (655–6550)	

* Calibrated.

† Assumed to be half of diagnosed PPH cases.

C-section, caesarean section; GDP, Gross domestic product; IU, international unit; KOL, key opinion leaders; mL, Milliliters; PHC, primary health centres; PPH, postpartum haemorrhage; USD, United States dollars.

We used data from a recent Cochrane Review for risk of PPH-related health outcomes.^2^ The health outcomes modeled were PPH with blood loss of ≥500 mL, severe PPH (blood loss of ≥1000 mL), additional uterotonic treatment, and blood transfusions. We extracted the outcomes specific to taking oxytocin with misoprostol, taking oxytocin only, and having no treatment for the prevention of PPH along with birth method (vaginal or c-section). Given varying practices and resources in obstetric facilities, not all cases of PPH are diagnosed, resulting in a difference between data on reported PPH cases and the actual risk of PPH. We accounted for this by estimating the proportion of PPH cases being diagnosed using a recent study conducted in Nigerian facilities that documented differences in obstetric practices and outcomes of PPH.^24^

We applied data obtained from the E-MOTIVE trial, a multicentre randomised trial, to estimate the health outcomes of mothers who received substandard uterotonics.^9^ The E-MOTIVE trial examined the clinical outcomes of women giving birth using a bundle of interventions including oxytocin for the prevention and treatment of PPH in secondary-level Nigerian hospitals.^9^ Data were obtained from 32 hospitals that participated in the trial across Nigeria between 1 August 2021 and 1 March 2022.^9^ The E-MOTIVE trial assessed the quality of oxytocin products used in these facilities and their relationship with PPH outcomes.^9^ Relative risks of PPH outcomes were analysed for patients in Nigeria who received poor-quality oxytocin compared with those who received quality-assured oxytocin, using a regression analysis that clustered at facilities and controlled for confounders including age, gestational age, parity, pregnancy type, mode of birth, birth weight, antepartum haemorrhage, pre-eclampsia, previous c-section, PPH in previous pregnancy, labour augmented or induced, retained placenta or manual removal of the placenta, episiotomy, and perineal tear. The relative risks were incorporated into the model to estimate the numbers of PPH and severe PPH cases for those who received substandard uterotonics. The probabilities of additional uterotonic use and blood transfusions were incorporated by calibrating probabilities to match the E-MOTIVE trial data in Nigeria.^9^

Finally, we located and contacted key informants in Nigeria to gather more information regarding expenditure linked to PPH in both public and private sectors, as well as the usage of blood products in different types of facilities. Cost inputs were collected through key informants covering 20 health facilities (four PHCs, seven public hospitals and nine private hospitals) in the North, and 12 facilities (one PHC, seven public hospitals and four private hospitals) in the South. Where available, data for north versus south Nigeria were entered separately due to the geographic, economic, and cultural differences between the regions and their impact on maternal health outcomes.^25 26^ The collected costs included OOP patient expenditures on treatment, hospitalisation, procedures and costs of blood products, and costs to provide PPH care from providers’ perspectives by levels of facilities. Treatment costs included the cost of medicines, where we assumed, without further evidence, that the price of substandard and quality uterotonics were the same. All costs were reported in 2022 United States Dollars (USD).

### Model structure

**Figure 1:**
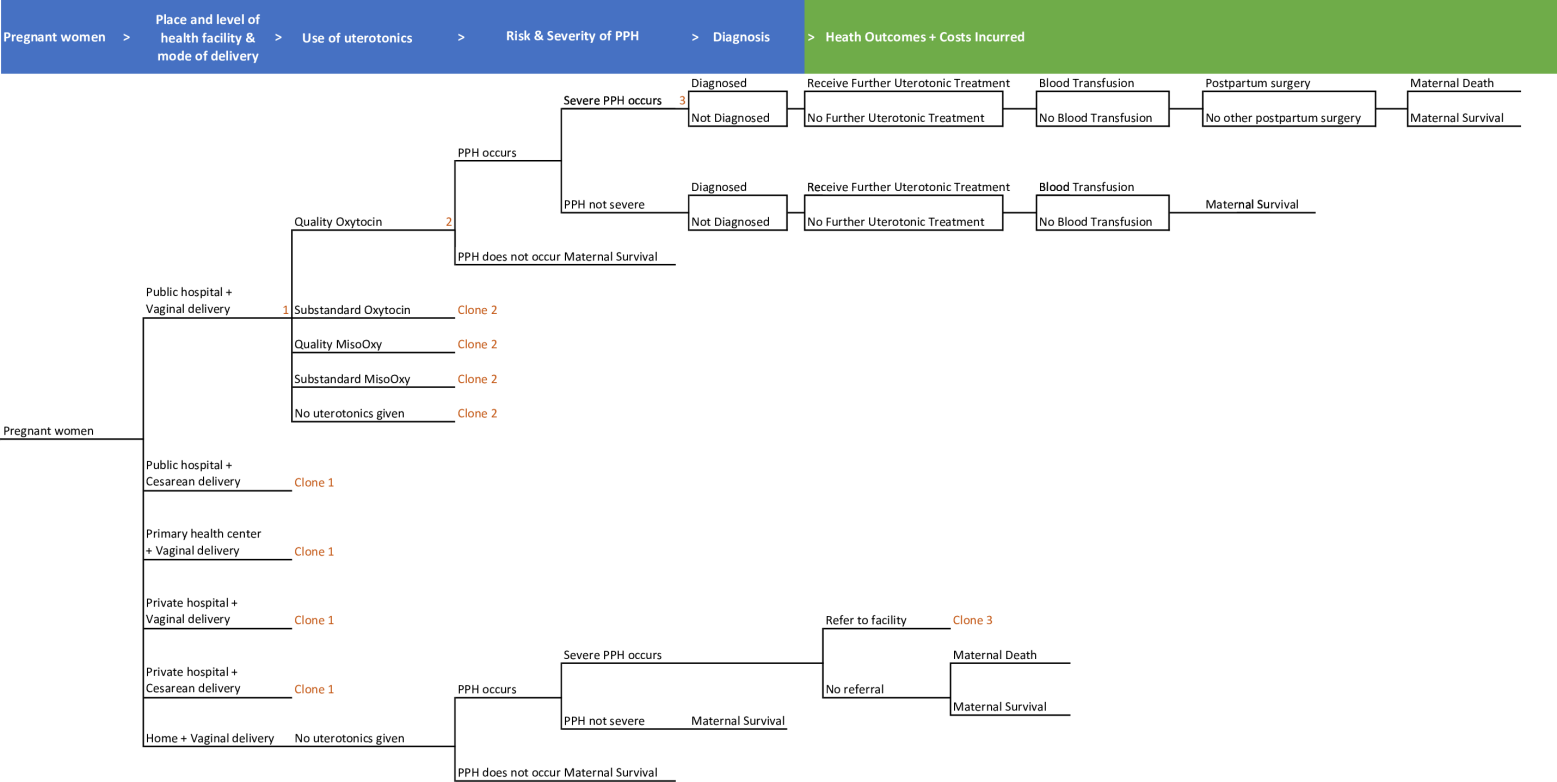
Model decision tree diagram. MisoOxy, misoprostol and oxytocin. PPH, postpartum haemorrhage.

We modelled the prophylactic use of uterotonics in the third stage of labour among Nigerian women giving birth. In figure 1, birthing women were simulated to deliver in public hospitals, PHCs, private hospitals, and their homes. The location and delivery method were simulated based on demographic characteristics. In the baseline scenario, mothers in health facilities were simulated to receive quality uterotonics, substandard uterotonics or no uterotonics to prevent PPH, while mothers giving birth at home did not use uterotonics. The uterotonic agents simulated in our baseline model included oxytocin alone, and oxytocin and misoprostol together. A proportion of women giving birth at home and at PHCs with severe PPH were referred to public hospitals. The health outcomes and subsequent steps such as blood transfusions, referrals to hospitals, and use of additional uterotonics were determined based on birth method (vaginal or c-sections), preventative uterotonic use, and the quality of preventative uterotonics.

### Model outputs

The primary model outputs are annual estimates of the health impact as well as the direct medical costs, and productivity losses attributable to the use of substandard uterotonics in the third stage of labour. Productivity losses refer to long-term productivity losses due to maternal deaths and were calculated using the gross domestic product per capita for the average years of life remaining for a mother who died of PPH, with future years discounted at 3%. The health impact is presented as the number of mothers with PPH (PPH≥500 mL) and severe PPH (≥1000 mL), counts of blood transfusions, and additional treatments, numbers of maternal deaths, and years of life lost (YLLs) from premature death. Direct medical costs consist of the costs of medications, blood transfusions, hospital stays, and other related procedures. We further break down the results by population characteristics including rurality, North/South region, and wealth quintile. We assumed that providers, including doctors, nurses, and auxiliary nurses, work 52 weeks per year and 40 hours per week. Costs to health providers were calculated using fractions of average monthly salaries that contribute to caring for patients with PPH. The time horizon and analysis timeframe of the model is 1 year. Model outputs are presented as annual averages with interquartile ranges (IQR).

### Model scenarios and sensitivity analysis

We modelled eight scenarios and described the details in the online supplemental figure S1–S6. In the first scenario, (1) the impact of substandard uterotonics in Nigeria was simulated by comparing the baseline, which used the reported prevalence of substandard uterotonics, with a scenario where all oxytocin and misoprostol were to be quality assured. Second, (2) maintaining baseline care-seeking patterns, we modelled a scenario where all births at facilities would use uterotonics by giving oxytocin to mothers at facilities who did not use any uterotonics in baseline. Third, (3) to further examine the impact of specific uterotonics, we simulated the use of a combination regimen of both quality misoprostol and oxytocin together in all facility births.

We also simulated a scenario of (4) using quality-assured heat-stable carbetocin, an alternative uterotonic recommended by WHO for the prevention of PPH. In this scenario, heat-stable carbetocin was simulated to be used in all facility births. We also simulated (5) a clinical trial-like situation where all women who had PPH delivered in a facility, all cases of PPH were diagnosed, and all medicines received were quality-assured. We then sought to (6) isolate the impact of uterotonic quality with a comparison scenario. This scenario assumed that all cases were diagnosed and delivered in facilities but changed all medicines to be substandard for comparison.

Moreover, previous efforts have demonstrated the potential of using misoprostol to prevent PPH in communities in Nigeria.^27 28^ We modelled two scenarios to scale up the use of misoprostol in home births, where we simulated the impact if misoprostol was to be provided to and used by all women giving birth at home. The first misoprostol scenario (7) used the reported level of substandard misoprostol found in the literature (33.7%), and another misoprostol scenario (8) simulated only the use of quality-assured misoprostol.^27 28^

We conducted sensitivity analyses where key data inputs were ranged probabilistically across 1000 model runs to account for natural variations in model inputs. The effectiveness of interventions varied based on beta distributions, while cost data were ranged using gamma distributions. Parameters that were varied in the probabilistic sensitivity analysis along with their uncertainty ranges are provided in table 1. Additionally, our study adheres to the Consolidated Health Economic Evaluation Reporting Standards, as outlined in online supplemental table S3.^29^

### Patients and public involvement

Our study design and data inputs were informed by guidance from our steering committee, which represented patient and public voices in Nigeria. We enlist the help of patients and the public in disseminating our research findings to influence policies and practices to improve the quality of uterotonics.

## Results

### Impact of PPH

We estimated that in Nigeria, approximately 29 000 (28 000–30 000) maternal deaths occur annually as a result of PPH (table 2). This translates to about 636 000 (612 000 to 663 000) YLLs and US$978 million in productivity costs (US$940 million to US$1 billion; ~₦770 billion) per year.^30^ Furthermore, costs of care for PPH in Nigeria added up to around US$1.3 billion (US$1.2 billion to US$1.3 billion; ~₦1 trillion) annually, which consisted of US$73 million (US$60 million to US$84 million; ~₦57.5 billion) OOP expenditures in the North, and US$220 million (US$190 million to US$246 million; ~₦173 billion) in the South.

The baseline results of the model estimate the total PPH impact in Nigeria while incorporating the reported levels of substandard uterotonics. Based on an estimate of 7.8 million pregnant women delivering in Nigeria per year, the baseline model simulated approximately 1.7 million (1.6–1.7 million) total cases of PPH, including around 366 000 (357 000–375 000) severe PPH cases. Among the PPH cases, the model estimated that around 176 000 (170 000–182 000) PPH cases were diagnosed, and of these 57 000 (54 000–59 000) were severe PPH cases diagnosed. Additional uterotonic treatments were estimated for 325 000 (317 000–334 000) cases, and blood transfusions were estimated to be required for 156 000 (151 000–160 000) PPH cases per year.

**Table 2:** Annual health and economic burden of postpartum haemorrhage in Nigeria comparing baseline to no substandard uterotonics

	Baseline	IQR	No substandard uterotonics	Difference	IQR of difference	Diff %
PPH≥500 mL, n	1 698 253	1 665 452–1 733 099	1 623 716	−74 536	−86 617 to −62 693	−4
PPH≥1000 mL, n	365 500	356 557–374 658	347 184	−18 316	−23 767 to −12 791	−5
PPH≥500 mL diagnosed, n	176 043	169 524–182 078	153 870	−22 173	−27 557 to −16 562	−13
PPH≥1000 mL diagnosed, n	56 549	54 402–58 508	51 064	−5484	−7 580 to −3 375	−10
Additional uterotonic treatment, n	325 363	316 821–333 758	258 792	−66 571	−72 741 to −60 482	−20
Blood transfusions, n	155 849	151 363–160 207	92 031	−63 817	−67 825 to −59 693	−41
Deaths due to PPH, n	28 669	27 557–29 846	27 215	−1454	−2 843 to −79	−5
YLL	636 453	611 755–662 588	604 173	−32 279	−63 104 to −1 753	−5
Total economic burden of PPH, USD	1 271 176 700	1 222 580 363–1 321 052 934	1 181 834 059	−89 342 640	−138 565 515 to −41 693 055	−7
Total OOP costs North, USD	73 176 618	60 158 794–83 702 431	63 751 328	−9 425 291	−12 399 356 to −5 909 113	−13
Total OOP costs South, USD	219 620 801	190 227 575–245 837 322	189 324 356	−30 296 445	−36 639 802 to −22 972 412	−14
Long-term productivity loss, USD	978 379 281	940 412 485–1 018 555 642	928 758 376	−49 620 905	−97 005 299 to −2 694 592	−5

Diff, difference; IQR, Interquartile range; mL, Milliliter; OOP, out-of-pocket; PPH, postpartum haemorrhage; USD, United States dollars; YLL, years of life lost.

### Impact of substandard uterotonics

The model estimated the economic burden of substandard uterotonics used during the third stage of labour to be US$89 million annually (~₦ 68.4 billion). Of this, we estimated US$9 million (~₦7 billion) was incurred as OOP costs in the North, US$30 million (~₦24 billion) as OOP costs in the South, and US$50 million (~₦39 billion) as long-term productivity losses due to deaths. Substandard uterotonics resulted in a 7% increase in overall costs, including a 14% increase in OOP costs, and a 5% increase in long-term productivity losses. In terms of the health outcomes driving these costs as compared with the baseline, we estimated that substandard uterotonics were responsible for around 75 000 of overall PPH cases, 18 000 of severe PPH cases, and 1500 maternal deaths due to PPH in Nigeria.

The results in table 3 detail the impact of different stakeholders on UHC in Nigeria. Taking the perspective of families, 1.6 million mothers, including 273 000 PPH cases, received substandard uterotonics annually. Use of substandard uterotonics incurred US$39.6 million (~₦ 30.4 billion) in OOP expenses to the patient and family for additional treatments, blood transfusions, and longer hospital stays. From the perspective of healthcare providers, we show that using high-quality uterotonics would reduce providers’ need to administer 483 000 doses of oxytocin and 59 000 units of blood products, which translates to the need to manage 64 000 fewer blood transfusion cases annually. This would save 214 000 hours of provider time annually, equating to the workload of 59 full-time doctors and 43.4 full-time nurses and midwives. This saved time is estimated to translate to cost savings of US$753 000 (~₦ 579 million) spent on salaries, which could be used to pay providers to expand care to more patients or offer more services. Using quality-assured uterotonics could lead to 16% and 20% fewer cases of PPH and severe PPH in Nigeria, respectively. Moreover, using quality uterotonics could avert approximately 5% of deaths due to PPH.

**Table 3:** Burden of substandard uterotonics by beneficiary in Nigeria

Perspective	Description	Estimate
Families	No. of mothers receiving substandard uterotonics	1 563 008
	No. of cases of postpartum haemorrhage receiving substandard uterotonics	273 412
	No. of cases of severe postpartum haemorrhage receiving substandard uterotonics	71 290
	Out-of-pocket costs from additional treatments, blood transfusions, and longer hospitalizations due to substandard uterotonics	US$ 39 619 345
	No. of maternal deaths averted by using quality uterotonics	1453
Healthcare Providers	No. of doses of oxytocin saved by using quality uterotonics	483 459
	Amount of blood units saved by using quality uterotonics	59 401
	Total hours of providers’ time saved by using quality uterotonics	214 456
	No. of full-time equivalents doctors not used	59
	No. of full-time equivalents nurses and midwives not used	43.4
	Annual costs of provider’s salaries saved by using quality uterotonics	US$ 752 918
	Costs of doctors’ salaries saved	US$ 560 701
	Costs of nurses and midwives’ salaries saved	US$ 192 217
	No. of blood transfusions averted by using quality uterotonics	63 605
Governments	% of postpartum haemorrhage cases receiving substandard uterotonics	16%
	% of severe postpartum haemorrhage cases receiving substandard uterotonics	20%
	% of maternal deaths averted by using quality uterotonics	5%

The annual burden of substandard uterotonics by mothers’ rurality, wealth quintile, and region in Nigeria are presented in the online supplemental table S4. While mothers in all categories were affected by the burden of substandard uterotonics, individuals accessing healthcare facilities to give birth faced much of the burden due the high prevalence of substandard medicines. We simulated that replacing substandard uterotonics with quality uterotonics would reduce PPH cases by 8% in urban areas and 2% in rural areas. Substandard uterotonics impacted 1%–11% of PPH cases across wealth quintiles. Improving uterotonic quality reduced PPH cases by 3% in the North and 9% in the South, saving US$30.6 million (~₦ 23.5 billion) in the North and US$58.6 million (~₦ 45 billion) in the South annually.

### Scenario analyses

Table 4 shows the results comparing the remaining scenarios with the baseline. We first compared scenarios using oxytocin, oxytocin with misoprostol, or heat-stable carbetocin with baseline. When quality oxytocin was given to mothers who previously did not receive a uterotonic in facilities (scenario 2: births at facilities all use quality uterotonics), 134 000 (8%) of PPH cases and 2000 deaths (7%) due to PPH were averted annually, decreasing total spending by US$130 million (~₦ 99 billion) (10%). Using a combination of both misoprostol and oxytocin (scenario 3: births at facilities all use quality oxytocin with misoprostol) showed a reduction of 199 000 PPH cases (12%) and US$187 million (~₦ 143 billion) (15%) in total expenditures. The use of heat-stable carbetocin in facility births (scenario 4: births at facilities all use quality heat-stable carbetocin) demonstrated a reduction in cases of PPH by nearly 189 000 (11%) and economic savings of US$175 million (~₦ 134 billion) (14%) per year compared with baseline.

**Table 4:** Estimated annual health and economic impact of improving uterotonic quality

	PPH≥500 mL	PPH≥1000 mL	Additional uterotonic treatment	Blood transfusion	Deaths due to PPH	Total economic burden by PPH	Total OOP costs	Long-term productivity losses
Baseline	1 698 253	365 500	325 363	155 849	28 669	US$ 1 271 176 700	US$ 292 797 419	US$ 978 379 281
1. No substandard uterotonics	1 623 716	347 184	258 792	92 031	27 215	US$ 1 181 834 059	US$ 253 075 684	US$ 928 758 376
Difference from baseline	−74 536	−18 316	−66 571	−63 817	−1454	US$ −89 342 640	US$ −39 721 735	US$ −49 620 905
% Difference	−4%	−5%	−20%	−41%	−5%	−7%	−14%	−5%
2. Births at facilities all use quality uterotonics	1 564 017	338 312	214 105	56 081	26 597	US$ 1 141 028 708	US$ 233 360 901	US$ 907 667 807
Difference from baseline	−134 236	−27 188	−111 258	−99 768	−2072	US$ −130 147 992	US$ −59 436 518	US$ −70 711 474
% Difference	−8%	−7%	−34%	−64%	−7%	−10%	−20%	−7%
3. Births at facilities all use quality oxytocin with misoprostol	1 499 048	330 808	184 342	49 712	25 892	US$ 1 084 235 161	US$ 200 613 890	US$ 883 621 271
Difference from baseline	−199 205	−34 692	−141 021	−106 137	−2777	US$ −186 941 538	US$ −92 183 529	US$ −94 758 010
% Difference	−12%	−9%	−43%	−68%	−10%	−15%	−31%	−10%
4. Births at facility all use quality heat-stable carbetocin	1 509 065	332 070	187 655	50 511	26 146	US$ 1 095 738 173	US$ 203 453 790	US$ 892 284 383
Difference from baseline	−189 188	−33 430	−137 708	−105 338	−2523	US$ −175 438 526	US$ −89 343 628	US$ −86 094 898
% Difference	−11%	−9%	−42%	−68%	−9%	−14%	−31%	−9%
5. All births happen at facilities: quality	1 086 389	269 549	831 088	278 777	21 215	US$ 1 074 653 367	US$ 350 659 705	US$ 723 993 662
Difference from baseline	−611 864	−95 951	505 725	122 929	−7454	US$ −196 523 333	US$ 57 862 286	US$ −254 385 619
% Difference	−36%	−26%	155%	79%	−26%	−15%	20%	−26%
6. All births happen at facilities: substandard	1 352 037	336 327	1 199 833	633 739	26 444	US$ 1 344 036 980	US$ 441 604 765	US$ 902 432 215
Difference from baseline	−346 216	−29 173	874 470	477 891	−2225	US$ 72 860 281	US$ 148 807 346	US$ −75 947 066
% Difference	−20%	−8%	269%	307%	−8%	6%	51%	−8%
7. Misoprostol used in home births at reported quality	1 250 119	326 609	316 450	147 227	25 643	US$ 1 166 317 437	US$ 291 192 214	US$ 875 125 224
Difference from baseline	−448 134	−38 891	−8913	−8622	−3026	US$ −104 859 262	US$ −1 605 205	US$ −103 254 057
% Difference	−26%	−11%	−3%	−6%	−11%	−8%	−1%	−11%
8. Quality misoprostol used in home births	1 170 715	306 388	312 176	142 815	24 038	US$ 1 110 948 187	US$ 290 598 622	US$ 820 349 565
Difference from baseline	−527 538	−59 112	−13 187	−13 033	−4631	US$ −160 228 513	US$ −2 198 796	US$ −158 029 716
% Difference	−31%	−16%	−4%	−8%	−16%	−13%	−1%	−16%

mL, Milliliter; OOP, out-of-pocket; PPH, postpartum haemorrhage.

In the clinical trial-like situation (scenario 5: all births happen at facilities—quality) where all cases are delivered in a facility, all cases of PPH are diagnosed, and all medicines received are quality assured, the number of medical procedures performed would increase, including administration of more uterotonic treatments and blood transfusions. This would increase overall OOP costs by US$58 million (~₦ 45 billion) (20%) but reduce 612 000 PPH cases annually. If all births in Nigeria happened in facilities where only quality-assured uterotonics were used, it could result in annual savings of US$197 million (15%) in total costs, including OOP expenses and productivity losses. Conversely, if substandard uterotonics were used and all deliveries happened in facilities (scenario 6: all births happen at facilities—substandard), total expenses would increase by US$73 million (~₦ 56 billion) (6%) annually. Using substandard uterotonics when all births are delivered at facilities increased OOP costs to be US$149 million (~₦ 115 billion) (51%) higher than the baseline, but reduced 346 000 PPH cases annually due to more care seeking.

When misoprostol at reported prevalence of substandard quality is used in home deliveries (scenario 7: misoprostol used in home births at reported quality), it was estimated that PPH cases could decrease by 26%, resulting in about 448 000 fewer PPH cases, 3000 lives saved and US$105 million (~₦ 80 billion) in annual savings. Moreover, if the quality of misoprostol distributed at home was assured (scenario 8: quality misoprostol used in home births), our model estimated that around 528 000 (31%) PPH cases can be prevented, and US$160 million (~₦ 123 billion) (13%) total expenditures can be avoided compared with baseline.

## Discussion

Our findings demonstrate that an estimated US$89 million (~₦ 68.4 billion) are wasted annually in Nigeria due to use of substandard uterotonics, including US$39 million (~₦ 29.9 billion) in OOP spending. Moreover, 75 000 PPH cases and 1500 maternal deaths due to PPH could be avoided if quality-assured uterotonics were made available. These findings confirm that substandard uterotonics in Nigeria are exacerbating the already significant burden PPH has on maternal mortality and morbidity in the country, and show that these poor health outcomes and costs are preventable. Continuing to allow substandard medicines to circulate in LMIC markets is costing families, health systems, and governments more in poor health outcomes and economic losses compared with when quality-assured medicines are used. The wide availability of substandard medicines is impeding LMIC countries’ progress towards SDG goals and UHC.

This is the first study to formally quantify the clinical outcomes and costs of PPH in a UHC context in Nigeria, and the role substandard uterotonics play in these costs and outcomes. Substandard uterotonics burden the health system and its actors, and result in preventable costs that could have been spent for better use. At the health system level, costs associated with equipment, consumables, other materials, as well as the time of healthcare providers required to manage a PPH emergency due to substandard uterotonics could be reallocated to other high-need areas of the health system, or reinvested into national health insurance schemes and processes. At the patient level, OOP expenses requiring families to purchase additional medicines, materials and blood for transfusions during a PPH emergency would be significantly reduced or avoided with quality uterotonics, reducing financial risks and burden on already stretched household budgets. Additionally, there are several long-lasting adverse outcomes that can result from PPH including increased maternal morbidity, neonatal complications, infection, anaemia, postpartum depression, and other mental health issues.^1^ All of these outcomes not only have a health impact on the mother and her family, but also come at an additional cost to the health system, which could have been reinvested towards meeting UHC goals by improving the quality of uterotonics.

Our findings underline the importance of mothers and healthcare providers having access to various quality-assured uterotonics for both prevention and treatment of PPH. This aligns with the published results of the E-MOTIVE trial, which showed that combined use of misoprostol and oxytocin as part of the WHO recommended bundle of treatments reduces occurrence of severe PPH by 60%, as well as result in observed reductions in blood loss, need for postpartum blood transfusions, and maternal deaths.^9^ Access to quality-assured misoprostol for home deliveries is also critical to reduce PPH occurrence, as an estimated 31% of PPH cases could be avoided if mothers have access to quality misoprostol when delivering at home, which would save an estimated US$160 million in total PPH costs per year.

Oxytocin is one of the most commonly used uterotonic for prevention and treatment of PPH. However, it is heat sensitive and must be stored at a temperature of 2–8°C. Proliferation of generic oxytocin and misoprostol products that are manufactured in LMICs, where manufacturers may be unable to invest in costly quality-assured manufacturing processes, combined with a lack of quality-assured storage and distribution processes, potentially pose threats to the quality of uterotonics circulating in LMIC markets. While the prevalence of substandard products has been well documented,^7 11^ and progress has been made in the past decade to increase the number of quality-assured uterotonics available in LMICs, quality issues with these products continue to exist and are compounded by a lack of functioning cold chains in many LMIC settings. Further investment on the part of the Nigerian government is needed to increase cold chain storage capacity, improve overall supply chain management, and promote training on adherence to clinical guidelines and good medicine storage practices among healthcare providers.^31 32^

Additionally, the emergence of heat-stable uterotonics such as heat-stable carbetocin that do not require cold chain storage could be considered by health policy-makers in some parts of Nigeria (eg. in the North) to better equip facilities to prevent PPH. The ongoing Research to Expand Access to Heat Stable Carbetocin (REACH) trial for PPH treatment will be essential to add to the body of evidence that could justify a switch to using heat-stable carbetocin.^33^ To this end, WHO’s 2017 and 2018 PPH-related recommendations provide alternatives or adjunct medicines—heat-stable carbetocin for prevention and tranexamic acid for treatment—that seek to address, in part, the challenge of assuring uterotonic quality by eliminating the impact of poor cold-chain and humidity on medication potency.

Our results are in line with other published studies on PPH burden in Nigeria in terms of estimates of cases and deaths. The estimated numbers of overall PPH and severe PPH cases are comparable with the E-MOTIVE trial’s estimates in Nigerian facilities with a prevalence of 18%–19% PPH with blood loss of ≥500 mL and 5% PPH with blood loss of ≥1000 mL among facility deliveries.^9^ The E-MOTIVE trial has further demonstrated that PPH cases are often underdiagnosed without facilities carefully weighing the amount of blood loss.^24^ We estimated that a total of 176 000 cases with PPH were diagnosed annually, which aligns with previous survey studies ranging between 2% and 3% of all deliveries reporting PPH.^24 34^ In addition, WHO estimated that there are 81 747 maternal deaths based on 7 806 000 live births in Nigeria in 2020.^1^ Among these maternal deaths in Nigeria, 23%–44% were attributable to PPH.^3^ We have confirmed that our estimates of PPH deaths fall within this range.

There are a number of limitations to our study. All models are limited by the quality and availability of data inputs. Specifically, country-specific information on potential risks of requiring additional uterotonic treatments, blood transfusions, and postpartum surgery, in relation to one another was limited. For those who did not receive uterotonics in our model, we used health outcome data of women receiving placebo instead of prophylactic uterotonics due to lack of data availability. There was a scarcity of data on the case fatality rate of PPH across various types of facilities and locations in the country, making it difficult to assess geographic distribution. Moreover, the scenarios examined changes in specific parameters in the Nigerian context, which may not have the same effect in different settings. To maximise the reproducibility of quantitative analyses, we rigorously reviewed the literature, used the best available data sources, and carefully calibrated the model with evidence from the E-MOTIVE trial where available. We validated our model inputs and necessary assumptions with key informants in the country and ran sensitivity analyses to capture the uncertainty around varying inputs.

Second, the prevalence of substandard uterotonics was also not possible to be assessed by rurality or region within Nigeria, where our estimates of burden in rural regions and in the North of Nigeria may be conservative. Furthermore, we were not able to capture the contribution of anaemia to PPH as data for clinical outcomes among anaemic birthing women in Nigeria were not available. Our scenario analysis also did not estimate how much it would cost the health system to implement changes such as distributing misoprostol for home births or switching to heat-stable carbetocin. Additional cost-effectiveness studies should be conducted considering the costs of misoprostol distribution or costs of any increased use of heat-stable carbetocin.^35^ Despite these limitations, we believe we have used the best available evidence to estimate the health and economic burden of substandard uterotonics in Nigeria.

Our findings demonstrate that use of substandard medicines is slowing down progress towards UHC. The study highlights the importance of safeguarding uterotonic medicine quality in Nigeria, where currently the maternal mortality ratio is 1047 maternal deaths per 100 000 live births. To bring this number down and meet the SDG target 3.1, SDG target 3.8, and UHC goals, uterotonic medicines must be of assured quality. Health ministries need to strengthen the governance of medicines procurement systems and invest in cold chain infrastructure for oxytocin, as well as ensure procurers are financially able and empowered to purchase quality-assured medicines as part of standard treatment guidelines that have been implemented at all levels of the health system. Additionally, investments in heat-stable uterotonics is an effective alternative to prevent excessive bleeding among pregnant women in under-resourced settings. Quality-assured uterotonics are essential to prevent haemorrhage, save mothers’ lives, avert preventable costs, and contribute towards UHC goals.

## Supplementary material

10.1136/bmjph-2023-000624 online supplemental file 1

## Data Availability

All data relevant to the study are included in the article or uploaded as supplementary information.
