# Supplementary material for: Modelling the economic impact of substandard uterotonics on postpartum haemorrhage in Nigeria: safeguarding medicine quality can reduce costs and contribute towards universal health coverage
Source: BMJ Public Health. 2025 Apr 5;3(1):e000624. doi: 10.1136/bmjph-2023-000624 (PMC11973754; doi:10.1136/bmjph-2023-000624)
Supplement: online supplemental file 1 [file bmjph-3-1-s001.docx]

**Modeling the economic impact of substandard uterotonics on postpartum hemorrhage in Nigeria: safeguarding medicine quality can reduce costs and contribute towards universal health coverage**

Yi-Fang Ashley Lee, Colleen R Higgins, Petra Procter, Sara Rushwan, Chimezie Anyakora, Ahmet Metin Gülmezoglu, Lester Chinery, Sachiko Ozawa

**Supplementary Materials**

Table of Contents

[Table S1. Additional model inputs and sources 2](#_Toc193913574)

[Table S2. Model inputs for provider salaries and service time 3](#_Toc193913575)

[Detailed descriptions of scenarios with selected decision tree diagrams for illustration* 4](#_Toc193913576)

[Figure S1. No substandard uterotonics: 4](#_Toc193913577)

[Figure S2. Births at facilities all use quality uterotonics: 4](#_Toc193913578)

[Figure S3. Births at facilities all use quality oxytocin with misoprostol: 5](#_Toc193913579)

[Figure S4. Births at facility all use quality heat-stable carbetocin: 5](#_Toc193913580)

[Figure S5. All births happen at facilities – quality/substandard: 6](#_Toc193913581)

[Figure S6. Misoprostol used in home births at reported quality/ Quality Misoprostol used in home births: 7](#_Toc193913582)

[Table S3. Consolidated Health Economic Evaluation Reporting Standards 2022 (CHEERS2022) Statement: Updated Reporting Guidance for Health Economic Evaluations 8](#_Toc193913583)

[Table S4. The annual burden of substandard uterotonics by mothers’ characteristics (rurality, wealth, North/South) in Nigeria 12](#_Toc193913584)

Table S1. Additional model inputs and sources

| **Parameter variable (Unit)** | **Value (Uncertainty range)** | | | | | | | **Source** |
| --- | --- | --- | --- | --- | --- | --- | --- | --- |
| **Mothers' Characteristics** | | | | | | | |  |
| **Wealth Quintile** | | **Urban** | | | **Rural** | | | Demographic and Health Survey^1^ |
| Poorest (%) | | 5 | | | 35 | | |  |
| Poorer (%) | | 10 | | | 31 | | |  |
| Middle (%) | | 21 | | | 20 | | |  |
| Richer (%) | | 32 | | | 10 | | |  |
| Richest (%) | | 32 | | | 4 | | |  |
| **Care-seeking locations and birth methods by mother's characteristics (%)** | | | | | | | | |
| **Rurality** | | **Urban** | | | **Rural** | | | Demographic and Health Survey^1^ |
| Public hospital and vaginal birth | | 19 | | | 8 | | |  |
| Public hospital and c-section | | 2 | | | 1 | | |  |
| Public PHC and vaginal birth | | 16 | | | 11 | | |  |
| Private hospital and vaginal birth | | 20 | | | 5 | | |  |
| Private hospital and c-section | | 3 | | | 0 | | |  |
| Home | | 39 | | | 75 | | |  |
| **Wealth Quintile** | | **Poorest** | **Poorer** | **Middle** | | **Richer** | **Richest** | Demographic and Health Survey^1^ |
| Public hospital and vaginal birth | | 9 | 15 | 28 | | 38 | 39 |  |
| Public hospital and c-section | | 0 | 0 | 1 | | 1 | 4 |  |
| Public PHC and vaginal birth | | 1 | 1 | 1 | | 1 | 1 |  |
| Private hospital and vaginal birth | | 1 | 4 | 10 | | 17 | 30 |  |
| Private hospital and c-section | | 0 | 0 | 1 | | 2 | 6 |  |
| Home (%) | | 88 | 79 | 60 | | 41 | 21 |  |
| **Region** | | **North** | | | **South** | | | Demographic and Health Survey^1^ |
| Public hospital and vaginal birth | | 19 | | | 37 | | |  |
| Public hospital and c-section | | 1 | | | 2 | | |  |
| Public PHC and vaginal birth | | 1 | | | 1 | | |  |
| Private hospital and vaginal birth | | 4 | | | 27 | | |  |
| Private hospital and c-section | | 0 | | | 4 | | |  |
| Home | | 75 | | | 30 | | |  |
| **Proportion diagnosed with PPH (%)** | | 30 | | | | | | Wakili et al.^2^ & Sotunsa et al.^3^ |

C-section = Caesarean section; PHC= primary health center; PPH= postpartum hemorrhage

Sources:

^1^ National Population Commission (NPC) [Nigeria] and ICF. Nigeria Demographic and Health Survey 2018. 2019. https://dhsprogram.com/pubs/pdf/FR359/FR359.pdf (accessed June 1, 2023).

^2^ Wakili AA, Aswat A, Timms R, *et al.* Differences in obstetric practices and outcomes of postpartum hemorrhage across Nigerian health facilities. *International Journal of Gynecology & Obstetrics* 2022; 158: 23–30.

^3^ Sotunsa J, Adeniyi A, Imaralu J, *et al.* Maternal near‐miss and death among women with postpartum haemorrhage: a secondary analysis of the Nigeria Near‐miss and Maternal Death Survey. *BJOG* 2019; 126: 19–25.

Table S2. Model inputs for provider salaries and service time

| **Monthly provider salary (USD)** | | |
| --- | --- | --- |
| Physician/Doctor | | |
| PHC | 603 | |
| Hospital | 724 | |
|  | | |
| Nurse and auxiliary nurse midwife | | |
| PHC | 267 | |
| Hospital | 326 | |
| **Time taken by physician/doctor for treatment (Minutes)** | | |
|  | Vaginal birth | C-section |
| Mild/moderate PPH at PHC | 129 | 0 |
| Mild/moderate PPH at hospital | 77 | 79 |
| Severe PPH at PHC | 0 | 0 |
| Severe PPH at hospital | 170 | 174 |
| **Time taken by nurse and auxiliary nurse midwife for treatment (Minutes)** | | |
|  | Vaginal birth | C-section |
| Mild/moderate PPH at PHC | 186 | 0 |
| Mild/moderate PPH at hospital | 56.5 | 78.5 |
| Severe PPH at PHC | 0 | 0 |
| Severe PPH at hospital | 109 | 93.5 |

Monthly provider salaries and service time were estimated by key opinion leaders.

C-section = Caesarean section; PHC= primary health center; PPH= postpartum hemorrhage; USD = United States dollars.

Detailed descriptions of scenarios with selected decision tree diagrams for illustration*

Figure S1. No substandard uterotonics:

In this scenario, all uterotonics are made to be quality assured while the probability of receiving a uterotonic remains the same as baseline. Receiving a quality uterotonic compared to a poor-quality one directly impacts the probability of PPH occurring, the probability of requiring additional treatment or blood transfusion, and indirectly the number of surgeries and maternal survivals.


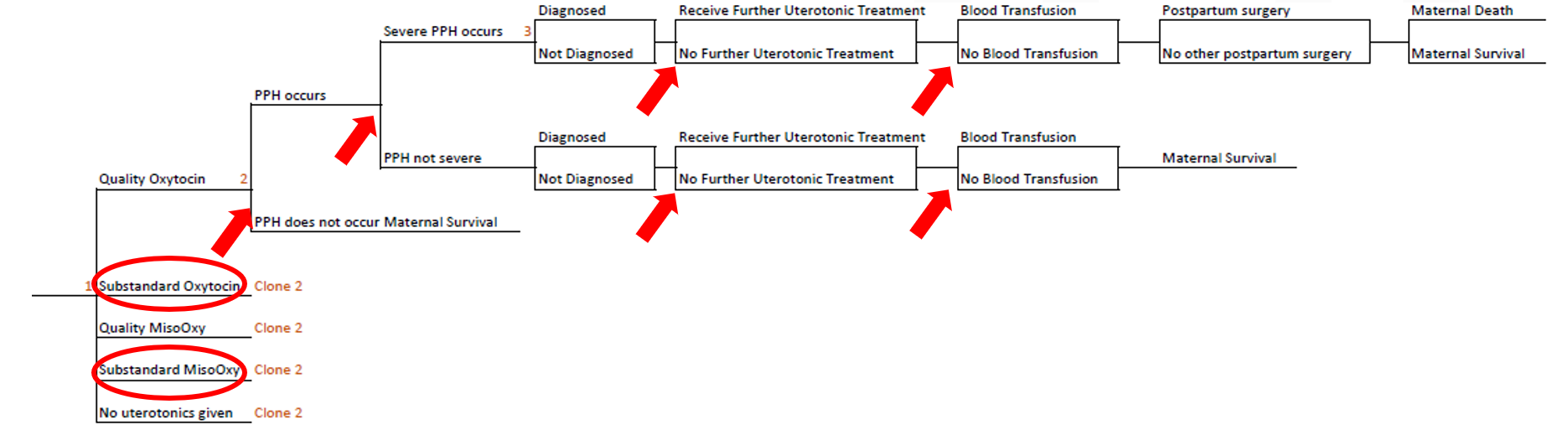


Figure S2. Births at facilities all use quality uterotonics:

In this scenario, utilization of uterotonics in facility births is increased. Those who receive no uterotonics in baseline instead receive oxytocin. This means more women are put into the quality and substandard oxytocin branches and face different probabilities of PPH occurring, the severity of PPH, and of receiving more treatment or blood transfusions, compared to the no uterotonics branch. This indirectly affects the numbers of surgeries and maternal survival.


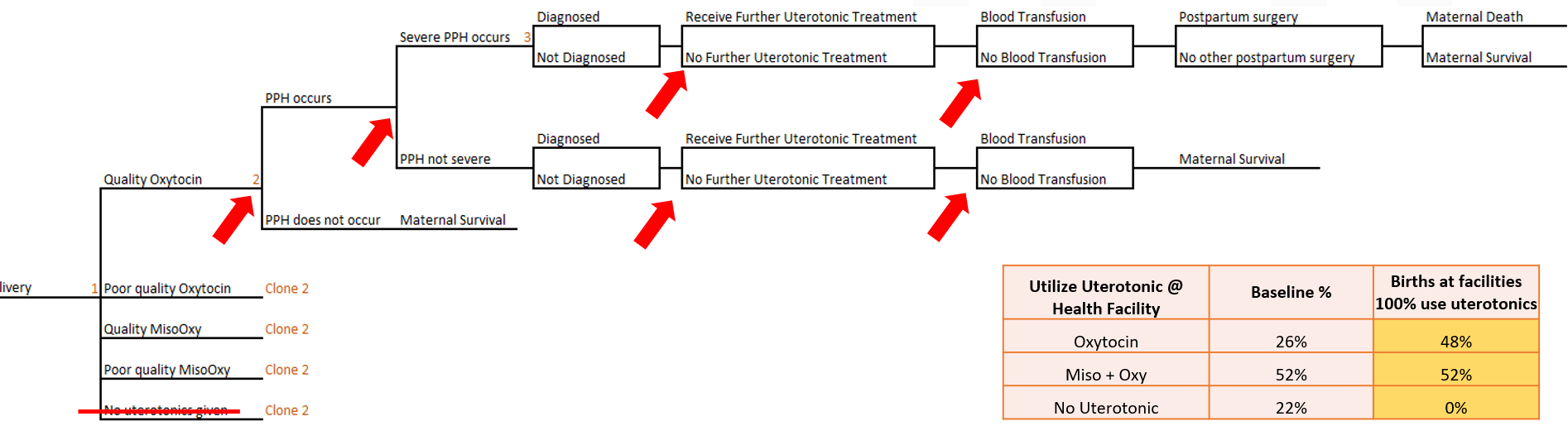


Figure S3. Births at facilities all use quality oxytocin with misoprostol:

In this scenario, all facility births are given misoprostol + oxytocin, and it is assumed to be quality-assured. This increases the number of women in the miso+oxy branch, where they face different probabilities of PPH occurring, severity of PPH, and of receiving further treatment or blood transfusions than they would in the oxytocin only branch. This then indirectly affects surgery, maternal survival or death.


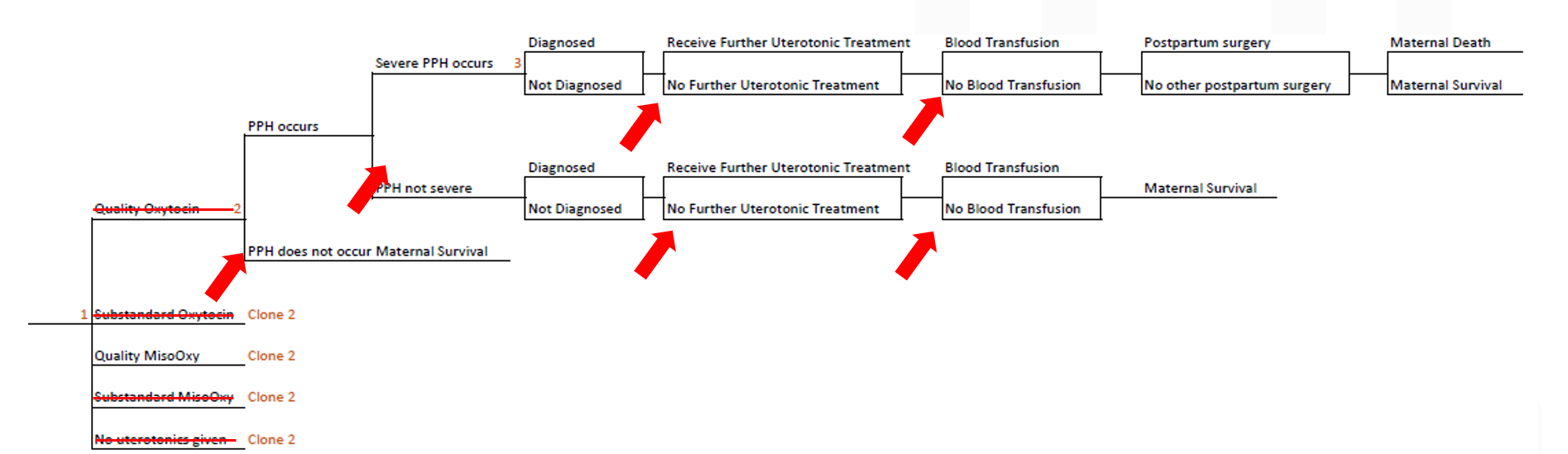


Figure S4. Births at facility all use quality heat-stable carbetocin:

In this scenario, carbetocin is the only uterotonic used and it is assumed to be quality-assured. This changes the probabilities of PPH occurring, the severity of PPH, and indirectly affects the probabilities of receiving more treatment, blood transfusions, surgery, and maternal survival or death.


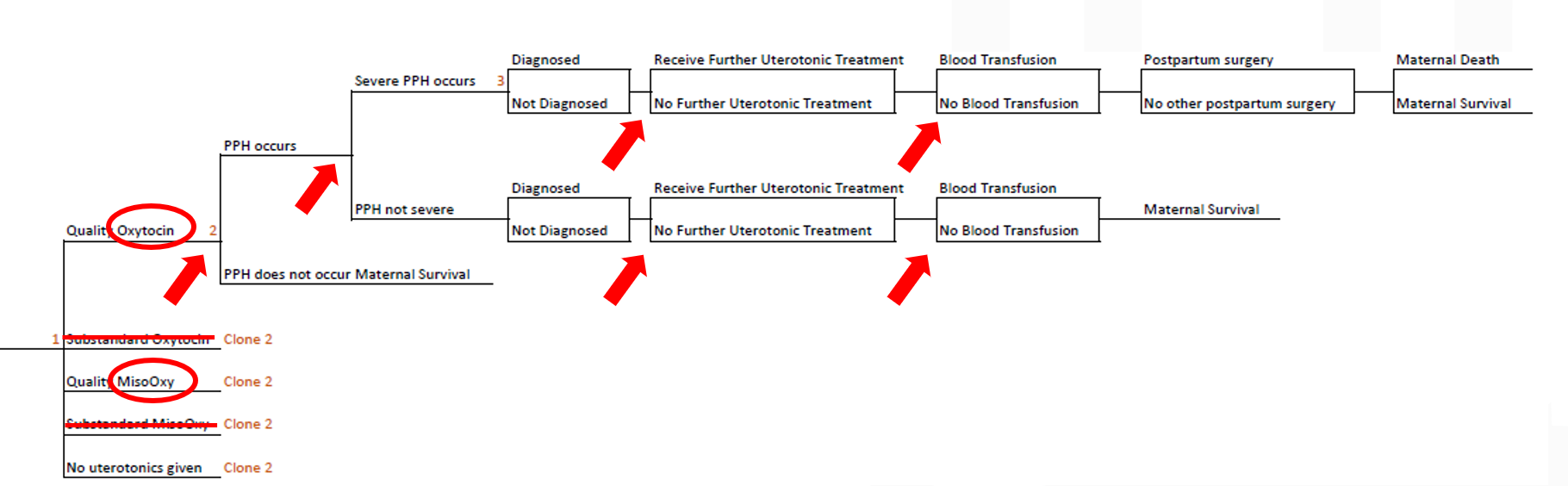


Figure S5. All births happen at facilities – quality/substandard:

In two scenarios, we show the impact of all births occurring at facilities. All births at facilities with quality uterotonics simulates a clinical trial-like situation where all cases of PPH are diagnosed and all medicines received are quality-assured. Then we turn all medicines substandard to isolate the impact of uterotonic quality.


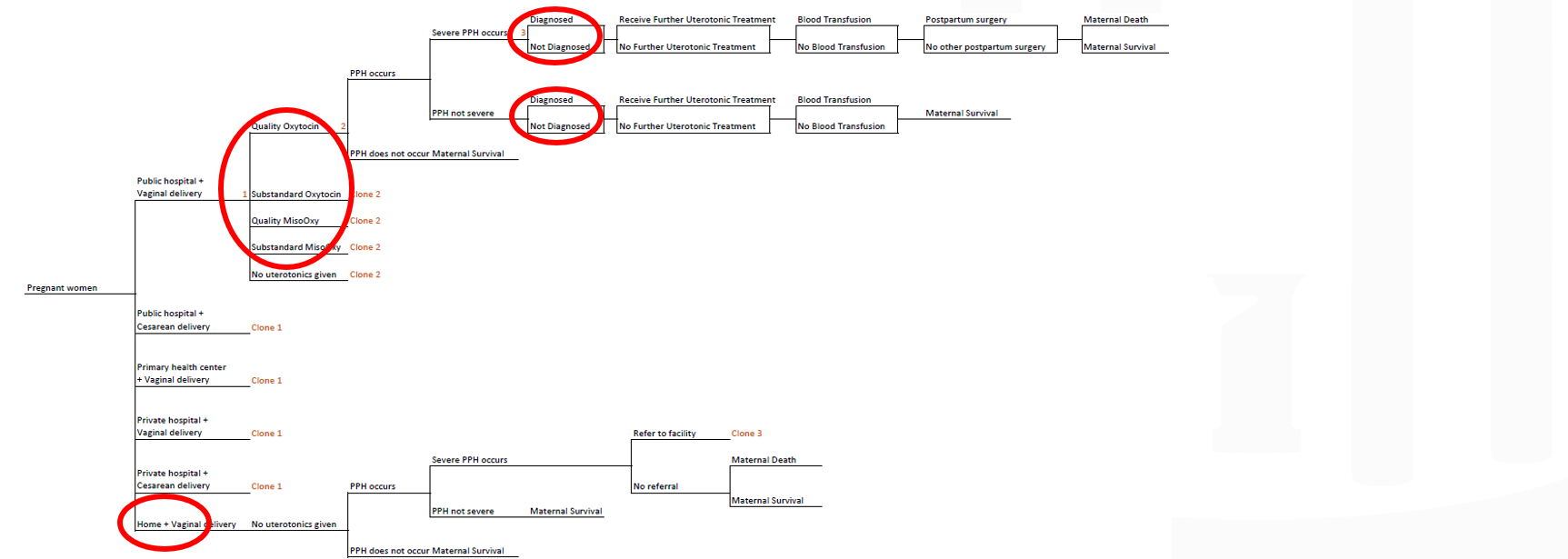


Figure S6. Misoprostol used in home births at reported quality/ Quality Misoprostol used in home births:

In these scenarios, misoprostol is provided and utilized by 100% of women giving birth at home in Nigeria. The quality of misoprostol at home is assumed to reflect the quality of misoprostol found in the literature in one scenario. We assumed only quality-assured misoprostol was utilized in another scenario. This directly impacts the probability of PPH in home births, and indirectly impacts the referral to a facility for further care and maternal survival.


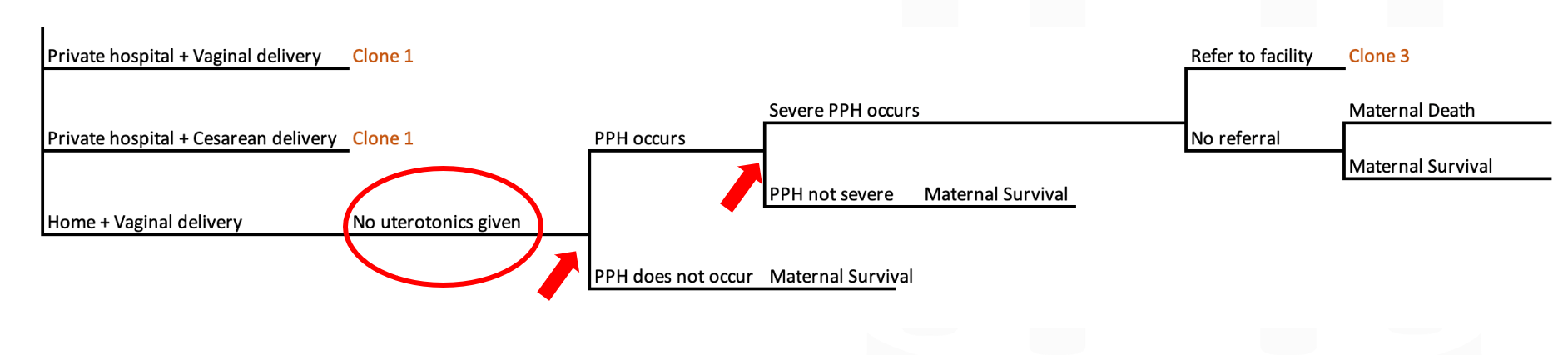


*Circles highlight where input changes were made in the described scenario. Arrows indicate the resulting impacts in the scenario. Horizontal lines represent the options being removed.

Table S3. Consolidated Health Economic Evaluation Reporting Standards 2022 (CHEERS2022) Statement: Updated Reporting Guidance for Health Economic Evaluations

# **CHEERS 2022 Checklist**

| **Topic** | **No.** | **Item** | **Location where item is reported** |
| --- | --- | --- | --- |
| **Title** |  |  |  |
|  | 1 | Identify the study as an economic evaluation and specify the interventions being compared. | Modelling study |
| **Abstract** |  |  |  |
|  | 2 | Provide a structured summary that highlights context, key methods, results, and alternative analyses. | Abstract |
| **Introduction** |  |  |  |
| **Background and objectives** | 3 | Give the context for the study, the study question, and its practical relevance for decision making in policy or practice. | Introduction, last paragraph |
| **Methods** |  |  |  |
| **Health economic analysis plan** | 4 | Indicate whether a health economic analysis plan was developed and where available. | Not Applicable |
| **Study population** | 5 | Describe characteristics of the study population (such as age range, demographics, socioeconomic, or clinical characteristics). | Model inputs,  Table 1, S1 |
| **Setting and location** | 6 | Provide relevant contextual information that may influence findings. | Model inputs |
| **Comparators** | 7 | Describe the interventions or strategies being compared and why chosen. | Model scenarios & sensitivity analysis |
| **Perspective** | 8 | State the perspective(s) adopted by the study and why chosen. | Model inputs |
| **Time horizon** | 9 | State the time horizon for the study and why appropriate. | Model outputs |
| **Discount rate** | 10 | Report the discount rate(s) and reason chosen. | Model outputs |
| **Selection of outcomes** | 11 | Describe what outcomes were used as the measure(s) of benefit(s) and harm(s). | Model inputs, outputs |
| **Measurement of outcomes** | 12 | Describe how outcomes used to capture benefit(s) and harm(s) were measured. | Model inputs, outputs |
| **Valuation of outcomes** | 13 | Describe the population and methods used to measure and value outcomes. | Model outputs |
| **Measurement and valuation of resources and costs** | 14 | Describe how costs were valued. | Model outputs |
| **Currency, price date, and conversion** | 15 | Report the dates of the estimated resource quantities and unit costs, plus the currency and year of conversion. | Model inputs |
| **Rationale and description of model** | 16 | If modelling is used, describe in detail and why used. Report if the model is publicly available and where it can be accessed. | Model structure |
| **Analytics and assumptions** | 17 | Describe any methods for analysing or statistically transforming data, any extrapolation methods, and approaches for validating any model used. | Model inputs, structure, outputs |
| **Characterising heterogeneity** | 18 | Describe any methods used for estimating how the results of the study vary for subgroups. | Model inputs, structure, outputs |
| **Characterising distributional effects** | 19 | Describe how impacts are distributed across different individuals or adjustments made to reflect priority populations. | Impact of substandard uterotonics, Table S4 |
| **Characterising uncertainty** | 20 | Describe methods to characterise any sources of uncertainty in the analysis. | Model scenarios and sensitivity analysis, Table 1 |
| **Approach to engagement with patients and others affected by the study** | 21 | Describe any approaches to engage patients or service recipients, the general public, communities, or stakeholders (such as clinicians or payers) in the design of the study. | Patients and public involvement |
| **Results** |  |  |  |
| **Study parameters** | 22 | Report all analytic inputs (such as values, ranges, references) including uncertainty or distributional assumptions. | Model inputs,  Table 1, S1 |
| **Summary of main results** | 23 | Report the mean values for the main categories of costs and outcomes of interest and summarise them in the most appropriate overall measure. | Impact of PPH, Impact of substandard uterotonics, Table 2, 3 |
| **Effect of uncertainty** | 24 | Describe how uncertainty about analytic judgments, inputs, or projections affect findings. Report the effect of choice of discount rate and time horizon, if applicable. | Scenario analysis, Table 2, 4 |
| **Effect of engagement with patients and others affected by the study** | 25 | Report on any difference patient/service recipient, general public, community, or stakeholder involvement made to the approach or findings of the study | Patients and public involvement |
| **Discussion** |  |  |  |
| **Study findings, limitations, generalisability, and current knowledge** | 26 | Report key findings, limitations, ethical or equity considerations not captured, and how these could affect patients, policy, or practice. | Discussion |
| **Other relevant information** |  |  |  |
| **Source of funding** | 27 | Describe how the study was funded and any role of the funder in the identification, design, conduct, and reporting of the analysis | Funding statement |
| **Conflicts of interest** | 28 | Report authors conflicts of interest according to journal or International Committee of Medical Journal Editors requirements. | Competing interests |

Table S4. The annual burden of substandard uterotonics by mothers’ characteristics (rurality, wealth, North/South) in Nigeria

| **PPH ≥500 ml** | ***Baseline*** | ***No substandard uterotonics*** | ***Difference*** | ***Proportion of reduction*** | ***Diff %*** |
| --- | --- | --- | --- | --- | --- |
| Overall | 1,698,253 | 1,624,462 | -73,790 |  | -4% |
| Urban | 612,734 | 565,896 | -46,838 | 63% | -8% |
| Rural | 1,085,519 | 1,058,566 | -26,952 | 37% | -2% |
| Poorest | 426,309 | 422,220 | -4,090 | 6% | -1% |
| Poorer | 403,136 | 395,660 | -7,476 | 10% | -2% |
| Middle | 339,226 | 324,791 | -14,434 | 20% | -4% |
| Richer | 281,049 | 261,028 | -20,021 | 27% | -7% |
| Richest | 248,533 | 220,764 | -27,769 | 38% | -11% |
| North | 1,209,183 | 1,177,162 | -32,022 | 43% | -3% |
| South | 489,070 | 447,301 | -41,769 | 57% | -9% |
| **PPH ≥1000 ml** |  |  |  |  |  |
| Overall | 365,500 | 347,488 | -18,012 |  | -5% |
| Urban | 138,875 | 127,511 | -11,364 | 63% | -8% |
| Rural | 226,625 | 219,977 | -6,648 | 37% | -3% |
| Poorest | 86,759 | 85,782 | -977 | 5% | -1% |
| Poorer | 83,249 | 81,376 | -1,873 | 10% | -2% |
| Middle | 72,938 | 69,369 | -3,569 | 20% | -5% |
| Richer | 63,234 | 58,339 | -4,895 | 27% | -8% |
| Richest | 59,319 | 52,622 | -6,698 | 37% | -11% |
| North | 252,998 | 245,162 | -7,836 | 44% | -3% |
| South | 112,501 | 102,326 | -10,175 | 56% | -9% |
| **Deaths due to PPH** |  |  |  |  |  |
| Overall | 28,669 | 27,216 | -1,453 |  | -5% |
| Urban | 10,912 | 10,008 | -905 | 62% | -8% |
| Rural | 17,757 | 17,209 | -548 | 38% | -3% |
| Poorest | 6,790 | 6,717 | -73 | 5% | -1% |
| Poorer | 6,496 | 6,387 | -109 | 8% | -2% |
| Middle | 5,743 | 5,404 | -339 | 23% | -6% |
| Richer | 4,961 | 4,575 | -386 | 27% | -8% |
| Richest | 4,678 | 4,133 | -545 | 38% | -12% |
| North | 19,818 | 19,198 | -620 | 43% | -3% |
| South | 8,851 | 8,018 | -833 | 57% | -9% |
| **Total economic burden of PPH** |  |  |  |  |  |
| Overall | $1,271,176,700 | $1,181,979,563 | -89,197,137 |  | -7% |
| Urban | $575,370,631 | $516,704,015 | -58,666,616 | 66% | -10% |
| Rural | $695,806,069 | $665,275,548 | -30,530,521 | 34% | -4% |
| Poorest | $243,318,574 | $239,571,905 | -3,746,669 | 4% | -2% |
| Poorer | $242,712,843 | $236,400,353 | -6,312,489 | 7% | -3% |
| Middle | $240,090,244 | $222,762,151 | -17,328,094 | 19% | -7% |
| Richer | $243,414,522 | $220,208,088 | -23,206,434 | 26% | -10% |
| Richest | $301,640,516 | $263,037,066 | -38,603,450 | 43% | -13% |
| North | $749,502,954 | $718,911,626 | -30,591,328 | 34% | -4% |
| South | $521,673,746 | $463,067,937 | -58,605,809 | 66% | -11% |
| **Total OOP costs** |  |  |  |  |  |
| Overall | $292,797,419 | $253,178,074 | -39,619,345 |  | -14% |
| Urban | $202,975,371 | $175,180,692 | -27,794,679 | 70% | -14% |
| Rural | $89,822,048 | $77,997,382 | -11,824,666 | 30% | -13% |
| Poorest | $11,583,692 | $10,340,299 | -1,243,394 | 3% | -11% |
| Poorer | $21,028,788 | $18,442,919 | -2,585,869 | 7% | -12% |
| Middle | $44,088,342 | $38,328,131 | -5,760,212 | 15% | -13% |
| Richer | $74,099,856 | $64,078,059 | -10,021,797 | 25% | -14% |
| Richest | $141,996,740 | $121,988,667 | -20,008,073 | 51% | -14% |
| North | $73,176,618 | $63,735,140 | -9,441,478 | 24% | -13% |
| South | $219,620,801 | $189,442,933 | -30,177,867 | 76% | -14% |
| **Long-term productivity losses** |  |  |  |  |  |
| Overall | $978,379,281 | $928,801,489 | -49,577,792 |  | -5% |
| Urban | $372,395,260 | $341,523,324 | -30,871,936 | 62% | -8% |
| Rural | $605,984,021 | $587,278,166 | -18,705,855 | 38% | -3% |
| Poorest | $231,734,882 | $229,231,606 | -2,503,276 | 5% | -1% |
| Poorer | $221,684,055 | $217,957,434 | -3,726,620 | 8% | -2% |
| Middle | $196,001,902 | $184,434,020 | -11,567,882 | 23% | -6% |
| Richer | $169,314,666 | $156,130,029 | -13,184,637 | 27% | -8% |
| Richest | $159,643,777 | $141,048,400 | -18,595,377 | 38% | -12% |
| North | $676,326,336 | $655,176,486 | -21,149,850 | 43% | -3% |
| South | $302,052,945 | $273,625,003 | -28,427,942 | 57% | -9% |

Diff = Difference; mL = milliliters; OOP = Out-of-pocket; PPH = postpartum hemorrhage.
